# Reclamation of Marine Chitinous Materials for the Production of α-Glucosidase Inhibitors via Microbial Conversion

**DOI:** 10.3390/md15110350

**Published:** 2017-11-07

**Authors:** Van Bon Nguyen, San-Lang Wang

**Affiliations:** 1 Department of Science and Technology, Tay Nguyen University, Buon Ma Thuot City 630000, Vietnam; bondhtn@gmail.com; 2 Department of Chemistry, Tamkang University, New Taipei City 25137, Taiwan; 3 Life Science Development Center, Tamkang University, New Taipei City 25137, Taiwan

**Keywords:** chitin, crab shells, α-glucosidase inhibitor, *Paenibacillus*, diabetes

## Abstract

Six kinds of chitinous materials have been used as sole carbon/nitrogen (C/N) sources for producing α-glucosidase inhibitors (aGI) by *Paenibacillus* sp. TKU042. The aGI productivity was found to be highest in the culture supernatants using demineralized crab shell powder (deCSP) and demineralized shrimp shell powder (deSSP) as the C/N source. The half maximal inhibitory concentration (IC_50_) and maximum aGI activity of fermented deCSP (38 µg/mL, 98%), deSSP (108 µg/mL, 89%), squid pen powder (SPP) (422 µg/mL, 98%), and shrimp head powder (SHP) (455 µg/mL, 92%) were compared with those of fermented nutrient broth (FNB) (81 µg/mL, 93%) and acarbose (1095 µg/mL, 74%), a commercial antidiabetic drug. The result of the protein/chitin ratio on aGI production showed that the optimal ratio was 0.2/1. Fermented deCSP showed lower IC_50_ and higher maximum inhibitory activity than those of acarbose against rat intestinal α-glucosidase.

## 1. Introduction

Chitin and its derivatives hold great economic value due to their versatile activities and biotechnological applications. Among the natural chitin-containing resources, crab shells, shrimp shells, and squid pens have the highest chitin content. Conventionally, chitin is obtained from crab shells and shrimp shells by using an inorganic acid or a strong alkali for demineralization or deproteinization, respectively. However, these chemical treatments have several drawbacks, such as the creation of pollutant acid or alkali liquid. Furthermore, the utilization of the deproteinized liquid bioresources is reduced because of the presence of an alkali [1].

Among the fishery chitinous materials, squid pens contain the highest ratio of protein [2]. For the recycling squid pens in order to produce additional highly bioactive products other than chitin or chitosan, the utilization of squid pens has been estimated via microbial conversion [3,4,5,6,7,8,9,10,11,12,13,14,15]. Many microorganisms produce chitinolytic and/or proteolytic enzymes using squid pens as the sole carbon/nitrogen (C/N) source [3,4,13]. These findings provide promising results for the production of chitin or chitin oligomers from this chitinous material. In addition, investigations have also included the production of enzymes [3,4,5], exopolysaccharides [6,7,8,9], chito-oligomers [3], antioxidants [10], insecticidal materials [11,12], biofertilizers [13], and biosorbents [14,15]. These chitinous materials were utilized for another purpose in the present study, the synthesis of antidiabetic drugs via microbial conversion.

Over 90% of diabetes mellitus (DM) cases are type 2 (non-insulin-dependent DM). The use of α-glucosidase inhibitors (aGI), such as acarbose, miglitol, and voglibose has been reported in regard to the treatment of type 2 diabetes. These remedies entailed some problematic side effects, such as diarrhea, flatulence, and abdominal discomfort. Therefore, there is an interest in discovering new natural sources of aGI. Until now, aGI have been reported to be produced by microorganisms [2,16]. Among these aGI-producing microbes, only strains of *Streptomyces* [17,18], *Bacillus* [19,20,21,22,23], *Stenotrophomonas maltrophilia* [24], and *Actinoplanes* spp. SE-50 [25] have been studied extensively. 

Many strains of *Paenibacillus* have been reported to use squid pens as the sole C/N source for producing exopolysaccharides [6,7,8,9]. Squid pens have been efficiently recycled via microbial fermentation to produce medicinal materials, and have also been used as the sole C/N source for the screening of aGI-producing bacterial strains. Our preceding studies revealed that *Paenibacillus* sp. TKU042, a bacterium isolated from Taiwanese soil, using squid pens as the sole C/N source, secreted acarbose-comparable aGI in the fermented nutrient broth. The optimization of culture conditions, pH and thermal stabilities, and the effect of fermented nutrient broth on mice were also explored [2].

In this study, six kinds of chitin-containing materials were used as the C/N source for aGI production by *Paenibacillus* sp. TKU042; other chitinolytic bacteria strains of *Paenibacillus* species were tested for aGI productivity. The effect of protein supplement and some cultivation parameters on the aGI productivity and the specific inhibition of *Paenibacillus* sp. TKU042 aGI were also investigated. Herein, the half maximal inhibitory concentration (IC_50_) and maximum activity were estimated and compared with those of fermented nutrient broth and acarbose.

## 2. Results and Discussion

### 2.1. Screening of Chitin-Containing Materials as C/N for α-Glucosidase Inhibitors Production

The effect of chitin-containing materials on aGI production by *Paenibacillus* sp. TKU042 was investigated with six kinds of chitinous materials: demineralized crab shell powder (deCSP), demineralized shrimp shell powder (deSSP), squid pen powder (SPP), shrimp head powder (SHP), fresh shrimp shell powder (frSSP), and cicada shell powder (CiSP) as the sole sources of C/N with the concentration of 1% (*w*/*v*). The protein–chitin–mineral salts compositions of shrimp shells and squid pen were 48%, 38%, and 14% and 61%, 38%, and 1%, respectively [26]. It is obvious that the mineral salts content in squid pen (1%, *w*/*w*) is much lower than in shrimp shell (14%, *w*/*w*). Therefore, only the crab shells and shrimp shells were decalcified and used as the C/N sources for aGI production.

**Figure 1:**
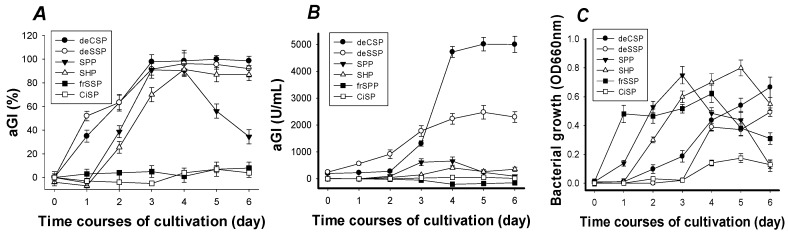
Screening of chitinous materials as the Carbon/Nitrogen (C/N) source for α-glucosidase inhibitors (aGI) production by *Paenibacillus* sp. TKU042. (**A**) percentage, (**B**) enzyme units per mL and (**C**) bacterial growth per cultivation time (day). deCSP: Demineralized crab shell powder; deSSP: Demineralized shrimp shell powder; SPP: Squid pen powder; SHP: Shrimp head powder; fSSP: Fresh shrimp shell powder; CiSP: Cicada shell powder. OD: optical density.

The results of maximum aGI activity, aGI productivity, and cell growth were investigated, and are shown in Figure 1. After four days of fermentation, the maximum aGI activity (Figure 1A) of most C/N sources reached the highest level, 91–100% with the priority sequence of deCSP, deSSP, SHP, and SPP. The maximum aGI activities of deCSP, deSSP, and SHP remained thereafter; only SPP decreased dramatically to a level lower than 40% on the 6th day. The reason might be related to the higher content (around 60%) of protein in SPP. It is also possible that an additional inhibitory component was made and accumulated with increased time that affected the activity of the active compound. The other possibility could be the decreased stability of the active ingredient in SPP after the fourth day. A similar phenomenon was also found in *Bacillus subtilis* TKU007, which showed that SHP was a better C/N source than SSP for the production of chitosanase and protease [27] and nattokinase [28]. Although the protein content of SPP was higher than that of SSP, the resultant enzyme productivity was not raised [27]. This might be related to the ratio of chitin and protein in the C/N sources.

The maximum aGI activity of frSSP and CiSP was lower than 20%, even though the culture time was lengthened to six days. The maximum aGI activity was speculated to be affected by the presence of high levels of mineral salts (in the case of frSSP and CiSP) or some additional factors that were eliminated during demineralization (in the case of deCSP and deSSP). As shown in Figure 1B, the aGI productivity reached its highest level after 4–5 days of fermentation. The highest was found in deCSP (5010 U/mL), deSSP (2476 U/mL), and then in the other four (SPP, SHP, frSSP, and CiSP) with aGI productivity lower than 660 U/mL. The same as the phenomenon of maximum activity found in Figure 1A, the aGI productivity of deCSP and deSSP remained steadily even after six days of fermentation.

To examine the relationship of aGI productivity and bacterial growth, the culture broth after removing the residual chitinous materials (C/N source) by centrifugation (Kubota 5922, Japan) at 150× *g* for 10 min was used for analyzing cell growth. As shown in Figure 1C, deCSP and deSSP were not the best C/N sources for cell growth. These results showed that the production of aGI may not have a direct relationship with cell growth.

The abovementioned comparisons of aGI production by *Paenibacillus* sp. TKU042 in different chitin-containing media are summarized in Table 1. The aGI activity of fermented nutrient broth (FNB) [2] and acarbose (an antidiabetic drug) were also compared. The aGI activities of the C/N sources all reached their highest level on the 4th day. The IC_50_ value was found in the priority sequence of deCSP (38 μg/mL), FNB (81 μg/mL), deSSP (108 μg/mL), SPP (422 μg/mL), SHP (455 μg/mL), and acarbose (1095 μg/mL). The maximum aGI activities were in the priority sequence of deCSP (98%), SPP (98%), FNB (93%), SHP (92%), SSP (89%), and then acarbose (74%). 

As for the productivity of aGI after fermentation under optimal conditions, deCSP showed the best yield of 26,316 kU/g, followed consecutively by FNB (12,346 kU/g), deSSP (9259 kU/g), SPP (2370 kU/g), and SHP (2198 kU/g). These tested chitinous materials were all potential C/N sources for aGI production by *Paenibacillus* sp. TKU042, due to their higher productivity than that of acarbose (913 kU/g).

The weight yields of the obtained culture supernatants were also compared. The weight yields of deCSP (2.03 g/L) and deSSP (1.60 g/L) were lower than that of FNB (6.5 g/L). These results showed that there were fewer contaminants and greater ease for further purification of the aGI from the culture supernatants of deCSP and deSSP than from that of FNB. These results showed that the deCSP and deSSP were the most remarkable C/N sources for aGI production by *Paenibacillus* sp. TKU042.

### 2.2. The Effect of Protein Supplement on α-Glucosidase Inhibitors Production

**Figure 2:**
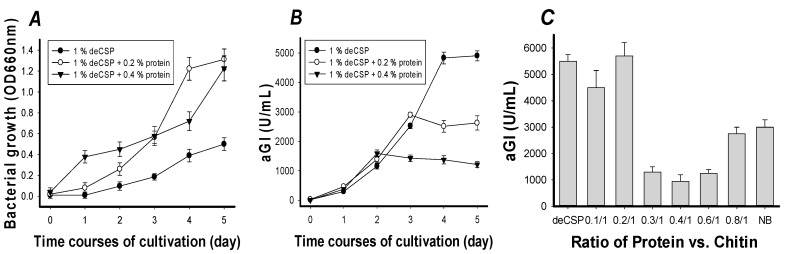
Effects of protein supplementation on the cell growth (**A**) and aGI production (**B**,**C**) of *Paenibacillus* sp. TKU042.

To analyze whether extra protein may enhance aGI production, the supplementation of 0.2% and 0.4% of protein (polypeptone/yeast extract = 4/6) in the deCSP (1%)-containing medium was studied. As shown in Figure 2, an inverse relationship between protein concentration and aGI productivity was found. The supplementation of free protein increased the bacterial growth (Figure 2A) but did not enhance the aGI productivity (Figure 2B). In the first two days, no significant difference in aGI productivity was found. The difference started to appear on the fourth day; the aGI productivity of these three C/N sources were in the order of 1% deCSP (4833 U/mL), 1% deCSP/0.2% protein (2512 U/mL), and then 1% deCSP/0.4% protein (1374 U/mL). According to the results, the ratio of protein/chitin appeared to play an important role in aGI production by *Paenibacillus* sp. TKU 042. The C/N sources (such as crab shell chitin or shrimp shell chitin) with chitin but only less than 1% of protein, were also found to be unsuitable for use as an inducer for the protease production by *B. subtilis* TKU007 [28] and *Bacillus* sp. TKU004 [29].

To investigate whether the protein in deCSP was sufficient to play an important role in aGI production by *Paenibacillus* sp. TKU 042, the protein in the deCSP was removed by heat-alkali-deproteinization treatment [29]. The obtained deproteinized deCSP was then used as the chitin sample for investigating the effect of ratio of protein vs. chitin (0.1/1–0.8/1) on aGI production. As shown in Figure 2C, the best results were found in the ratio of protein vs. chitin of 0.2/1 (5700 U/mL) and 0.1/1 (4500 U/mL). The optimal ratio of protein vs. chitin (0.2/1) investigated in this study was approximately the same as the deCSP, which showed comparable aGI productivity of 5500 U/mL. Another source of chitin (chitin obtained from SPP) was fermented by *Paenibacillus* sp. TKU 042 at the same ratio of protein vs. chitin and cultivation conditions as above. Similarly, this bacterium strain showed the same manner of aGI production with aGI productivity of 5600 U/mL. 

Nutrient broth (NB) containing proteins of polypeptone and yeast extract showed lower aGI productivity (3000 U/mL) than the ratio of protein vs. chitin (0.2/1), deCSP, and ratio of protein vs. chitin (0.1/1). These results showed that the supplementation of protein vs. chitin with a ratio higher than 0.2/1 decreased the aGI production. Therefore, deCSP was chosen for further optimization study.

### 2.3. Production of aGI from Demineralized Crab Shell Powder by Different Bacteria 

To examine whether other bacteria strains, especially the strains of *Paenibacillus* species, would also produce aGI in the deCSP-containing medium, 16 stocked chitinolytic bacteria which were all isolated from Taiwanese soils were tested. As shown in Table 2, only the bacteria of *Paenibacillus* species produced aGI against the tested three α-glucosidases. The maximum aGI activity against α-glucosidases of *Saccharomyces cerevisiae* (S), *Bacillus stearothermophilus* (B), and rat (R) were all above 90%.

Many strains of *Paenibacillus* species have been reported to be potentially used in agricultural, industrial, health food, and medical utilizations, such as the biosynthesis of antifungal agents [30,31], biofertizlizers [32,33], enzymes [22,24,25,26]. Recently, *Paenibacillus* species have also been explored to use SPP as the sole C/N source for the production of exopolysaccharides [6,7,8,9], antimicrobial biosurfactants [7], antioxidants [6], and homogentisic acid [16,34]. *Paenibacillus* sp. TKU042 was then chosen as the aGI-producing strain for further investigation.

### 2.4. Optimization of Culture Conditions

The deCSP was confirmed as a potential C/N source and chosen for an optimization study of some parameters, including the cultivation temperature (25, 30, 34, and 37 °C), culture volume (50, 70, 100, 130, and 160 mL), the seed culture volume (0.5, 1, 2, and 4 mL), and the concentration of deCSP (0.5, 0.75, 1, 1.25, 1.6, and 2%) (the data are shown in Supplementary Figure S1). Overall, aGI were effectively produced by *Paenibacillus* sp. TKU042 within the 1.6% deCSP-containing medium (130 mL medium in 250 mL-Erlenmeyer flask) at 30 °C in a reciprocal shaker (Yi Der LM-570R, Jun Yang, New Taipei City, Taiwan) at 150 rpm for four days (Figure S1). The culture conditions before and after the optimization study are summarized in Table 3. After the optimization was studied, the aGI productivity was increased from 5000 to 12,400 U/mL (2.48-fold), and the IC_50_ value was reduced from 81 to 6.7 µg/mL (12.1-fold). The culture supernatant (fermented deCSP) obtained by culturing *Paenibacillus* sp. TKU 042 in the optimized condition was then used for further study.

### 2.5. Specific α-Glucosidase Inhibitors Activity of Fermented Demineralized Crab Shell Powder

To evaluate the potential of *Paenibacillus* sp. TKU 042 aGI to be developed as antidiabetic drugs, the inhibitory specificity of fermented deCSP was tested against six kinds of commercial enzymes, including α-glucosidases (*S. cerevisiae*, *B. stearothermophilus*, rice, rat intestine) and α-amylases (*B. subtilis*, porcine pancreas).

As shown in Table 4, fermented deCSP showed lower IC_50_ (15.9 µg/mL) and higher maximum inhibitory activity (97%) than those of acarbose (78 µg/mL, 91%) against rat intestinal α-glucosidase. The IC_50_ and maximum inhibitory activity against *S. cerevisiae* α-glucosidase of fermented deCSP (6.7 µg/mL, 99%) were also better than those of acarbose (1095 µg/mL, 74%). Acarbose showed better results of IC_50_ (0.042 µg/mL and 3.04 µg/mL) against α-glucosidases from *B. stearothermophilus*, and rice, respectively, compared to fermented deCSP (6.6 µg/mL, 6.7 µg/mL). No inhibitory activity was found for fermented deCSP against porcine α-amylase and *B. subtilis* α-amylase. For the evaluation of the potential inhibitors using as antidiabetic drugs, α-glucosidase from rat has been suggested as the most valuable resource among the tested enzymes, since it is a mammalian enzyme closer to that of human [2]. In this study, fermented deCSP showed stronger inhibition against rat α-glucosidase than that of acarbose. This result suggested that fermented deCSP may be a good candidate of antidiabetic drugs. 

### 2.6. Confirmation of α-Glucosidase Inhibitors Containing in Fermented Chitin-Containing Media

**Figure 3:**
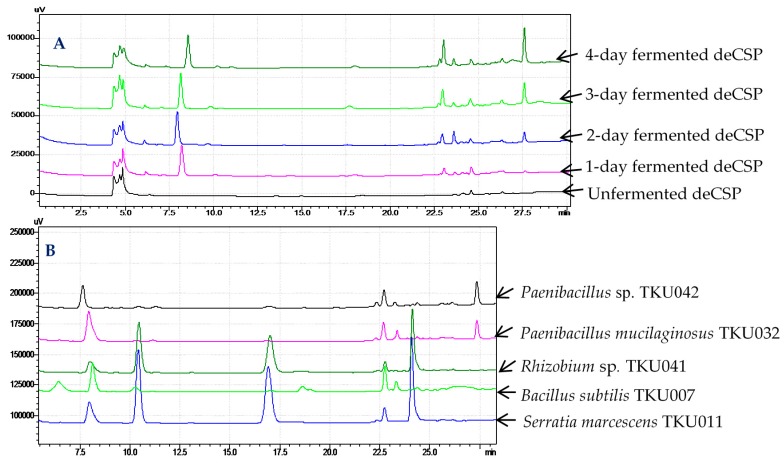
High-performance liquid chromatography (HPLC) fingerprints of (**A**) crab shell powder (CSP) fermented by TKU042 with different time courses of cultivation and (**B**) CSP fermented by various bacteria in four days.

The same concentration (20 mg/mL) of the unfermented deCSP and 1–4 day-fermented deCSP solutions were analyzed by high-performance liquid chromatography (HPLC). The difference of the HPLC fingerprints of deCSP before and after fermentation can be clearly observed in Figure 3A. After fermentation, some new main peaks appeared at the retention time of 8–8.5, 23, and 27.5 min, or enhanced its area (the peak at the retention time of 27.5 min). Unfermented deCSP was tested for α-glucosidase inhibition, but no activity was observed. The differences in HPLC fingerprints and the inhibitory activity between unfermented and fermented deCSP led to the conclusion that the aGI were produced by fermentation and had not previously existed in the deCSP.

To further compare which peaks especially belong to *Paenibacillus* sp., the four day-fermented deCSP by *Paenibacillus* sp. TKU042, *Paenibacillus mucilaginosus* TKU032, *Rhizobium* sp. TKU041, *B. subtilis* TKU007, and *Serratia marcescens* TKU011 were analyzed. As shown in Figure 3B, only the peak at the retention time of 27.5 min was found in deCSP fermented by *Paenibacillus* sp. TKU042 and *P. mucilaginosus* TKU032. Combining these results with the data shown in Table 2, this peak may signify the active compounds of aGI. The isolation and identification of these aGI will be performed soon.

## 3. Materials and Methods

### 3.1. Materials

Crab shells, shrimp shells, and squid pens were acquired from Shin-Ma Frozen Food Co. (I-Lan County, Taiwan). Fresh shrimp shells were prepared by drying them by lyophilization. Shrimp head powder was obtained from Fwu-Sow Industry (Taichun, Taiwan). Cicada shells were collected from the campus of Tamkang University (New Taipei, Taiwan). Demineralized crab shells and decalcified shrimp shells were prepared via acid treatment [29]. Nutrient broth was purchased from Creative Life Science Co. (Taipei, Taiwan). Rat α-glucosidase (intestinal acetone powders from rat) was purchased from Sigma Aldrich (St. Louis, MO, USA). Acarbose, *S. cerevisiae* α-glucosidase, *B. stearothermophilus* α-glucosidase, and 2,2-diphenyl-1-picrylhydrazyl (DPPH) were purchased from Sigma Aldrich (Singapore). Rice α-glucosidase (type 4) and porcine pancreatic α-amylase (type VI-B) were purchased from Sigma Aldrich. Some reagents, solvents, and other common chemicals were available at the highest grade.

### 3.2. Biological Assays of Enzymatic Inhibition

Enzyme inhibitory activity was modified from the method of Kwon et al. [35]. Fifty microliters of the sample solution were mixed with the same volume of the α-glucosidase solution and 100 μL of potassium phosphate buffer, and kept at 37 °C for 20 min. The addition of 50 μL of the substrate *p*-nitrophenyl glucopyranoside (*p*NPG) started the reaction, and this step was maintained at 37 °C for 40 min. One hundred microliters of a 1 mol/L Na_2_CO_3_ solution was added to stop the reaction. The final mixture solution was measured at 410 nm. The inhibition was calculated by using the following formula:

Inhibition (%) = (*A* − *B*)/ *A* × 100

where *A* is the absorbance at 410 nm of the reaction blank (no inhibitor/sample), and *B* is the absorbance at 410 nm of the reaction in the presence of the inhibitor/sample. The concentration of an inhibitor that can inhibit 50% of enzymatic activity under the assay conditions was defined as the IC_50_ value. The potassium phosphate buffer used was at a concentration of 0.1 mol/L, pH 7; this buffer was used for preparing enzymes, the sample, and the substrate solutions. *S. cerevisiae, B. stearothermophilus*, and rice α-glucosidases were used at concentrations of 0.25, 0.10, and 1.0 U/mL, respectively. The preparation of rat intestinal α-glucosidase solution was described in detail in a previous report [2]. The Statistical Analysis Software (SAS) version 9.4 (provided by SAS Institute Taiwan Ltd., Minsheng East Road, Section 2, Taipei, Taiwan 149-8) was employed to analyze the differences between the means of the calculated IC_50_ (µg/mL) and maximum inhibition (%) values at *p* < 0.01. 

### 3.3. The Effects of Chitin-Containing Materials and the Protein Supplement on aGI Production Experiments

Various kinds of chitin-containing materials, such as deCSP, deSSP, SPP, SHP, frSSP, and CiSP (*w*/*v*) were used as the sole sources of C/N. The cultural medium conducted in this study contains 0.1% K_2_HPO_4_, 0.05% MgSO_4_·7H_2_O, and 1% chitin-containing materials. The deCSP, the material for aGI production with the most potential, was chosen for the following experiments (The effects of the protein supplement on aGI production). Amounts of 0.2% and 0.4% protein (polypeptone/yeast extract = 4/6) were supplemented to the medium containing 1% deCSP. The above fermentation processes were performed at 30 °C, 150 rpm (shaking speed), 100/250 mL (ratio of volume of medium/Erlenmeyer flask), and the seed culture inoculated was 1 mL (OD_660nm_ = 0.25). The culture supernatants obtained by centrifugation at 500 rpm in 10 min to remove all the residues of chitinous materials were used to detect bacterial growth (measuring OD_660nm_), and the supernatants obtained by centrifugation at 12,000× *g* in 20 min to remove bacteria were conducted for Biological assays of enzymatic inhibition. The bacterial growth and inhibitory activity were detected daily; α-glucosidase from *S. cerevisiae* was used in the test, and the activity was expressed as % and U/mL. To investigate the optimal ratio of protein/chitin, the protein contained in the deCSP was removed by the method described in a previous study [29]; 0.1–0.8% free protein (polypeptone/yeast extract = 4/6) was then supplemented to the medium containing 1% chitin. The cultivation was performed as described above in four days; the supernatants obtained by centrifugation at 12,000 × *g* in 20 min were then used in the inhibition testing.

### 3.4. Optimization of Some Parameters for α-Glucosidase Inhibitors Productivity Production

Some major parameters, including the cultivation temperature (25, 30, 34, and 37 °C), culture volume (50, 70, 100, 130, and 160 mL), the concentration of deCSP (0.5%, 0.75%, 1%, 1.25%, 1.6%, and 2%), and the bacterial seed culture volume (0.5, 1, 2, and 4 mL) were considered for examination. The cultivation temperature experiments were conducted in a 250 mL Erlenmeyer flask with 100 mL of medium (initial pH 6.85) containing 0.05% MgSO_4_·7H_2_O, 0.1% K_2_HPO_4_, and 1% deCSP. Fermentation was performed in an incubator at the temperature range of 25–37 °C, with a shaking speed of 150 rpm for 4–5 days. The following experiments were designed based on the optimal conditions achieved from previous experiments. The *S. cerevisiae* α-glucosidase inhibition of the supernatants was tested and expressed as U/mL.

### 3.5. Conditions of High-Performance Liquid Chromatography Fingerprints Analysis

High-performance liquid chromatography fingerprints were analyzed under optimal conditions of mobile phase—0–5’ (0.1–1% methanol (MeOH)), 5–10’ (1–2% MeOH), 10–15’ (2–30% MeOH), 15–25’ (30–70% MeOH), and 25–35’ (70–100% MeOH)—and detected at 254 nm (ultraviolet detector), 0.6 mL/min (flow rate), and 25 °C (column temperature) using the C18 column.

## 4. Conclusions

Decalcified crab shells as the sole C/N source showed the best results for producing aGI compared to other tested chitin-containing materials by *Paenibacillus* sp. TKU042. The obtained culture supernatant showed higher aGI activity, lower IC_50_, and higher maximum inhibitory activity than those of acarbose. Among the tested 16 chitinolytic enzyme-producing bacteria, only the bacteria of *Paenibacillus* species produced aGI in the deCSP-containing medium. When the ratio of protein/chitin content in the liquid culture medium was adjusted to 0.2/1, the aGI activity obtained after fermentation was similar to that of decalcified crab shells. HPLC fingerprints showed the aGI produced by fermentation. All of these results suggest that chitin is a potential C/N source for producing aGI using *Paenibacillus* species. Furthermore, the culture supernatants might be useful candidates for treating type 2 diabetes. For evaluating the feasibility of commercialization of antidiabetic drugs using crab shells with a protein/chitin ratio of 0.2/1.0 and the bacteria of *Paenibacillus* species, the therapeutic effect ascertained in animal testing and the safety of human health when exposed to the aGI materials remains a challenge and requires a breakthrough.

## Figures and Tables

**Table 1:** Half maximal inhibitory concentration (IC_50_), maximum inhibition activity, and activity yield of the fermented chitinous materials (FCMs).

FCMs	Cultivation Time (Day)	Yield of Production (kU/g) ^a^	α-Glucosidase Inhibition	
			IC_50_ (µg/mL)	Maximal Inhibition (%) ^b^
deCSP	4	26,316	38 ± 4.1	98 ± 3.7
deSSP	4	9259	108 ± 5.2	89 ± 3.6
SPP	3	2370	422 ± 19	98 ± 1.9
SHP	4	2198	455 ± 42	92 ± 4.3
FNB **^c^**	4	12,346	81 ± 4.3	93 ± 4.2
acarbose **^d^**		913	1095 ± 93	74 ± 3.4

**^a^** The cultures of FCMs were harvested at 3–4 days of cultivation and centrifuged (Kubota 5922, Japan) at 12,000× *g* for 20 min to collect the culture supernatants. The supernatants were then dried by lyophilization and used to determine the yield of productivity and expressed as (kU/g). **^b^** The samples (FCMs and fermented nutrient broth (FNB)) and acarbose were tested at the concentration ranges of 15.6–1000 and 650–5000 µg/mL, respectively. **^c^** The FNB was obtained from a previous study [2]. **^d^** Acarbose, a commercial aGI, was used as a positive control.

**Table 2:** Comparison of the maximum inhibitory activity of α-glucosidase inhibitors produced by various bacteria using deCSP * as the C/N source.

No.	Bacterial Strain	Sources of α-Glucosidase		
		S	B	R
1	*Bacillus licheniformis* TKU004	-	-	-
2	*Bacillus subtilis* TKU007	-	-	-
3	*Bacillus mycoides* TKU038	-	-	-
4	*Bacillus mycoildes* TKU040	-	-	-
5	*Paenibacillus macerans* TKU029	96	90	92
6	*Paenibacillus mucilaginosus* TKU032	98	95	95
7	*Paenibacillus* sp. TKU037	99	95	90
8	*Paenibacillus* sp. TKU042	99	97	96
9	*Serratia marcescens* TKU011	-	-	-
10	*Serratia ureilytica* TKU013	-	-	-
11	*Serratia marcescens* TKU019	-	-	-
12	*Lactobacillus paracasei* TKU010	-	-	-
13	*Pseudomonas tamsuii* TKU015	-	-	-
14	*Serratia* sp. TKU016	-	-	-
15	*Serratia* sp. TKU020	-	-	-
16	*Rhizobium* sp. TKU041	-	-	-
Control (medium only)		-	-	-

* deCSP was used as the sole sources of C/N with concentrations of 1%. After four days of fermentation, the supernatants (50 μL) were then tested for their inhibition activity against *Saccharomyces cerevisiae* (**S**), *Bacillus stearothermophilus* (**B**), and rat intestinal (**R**) α-glucosidases (100 μL) using the assay mentioned in the Materials and Methods section, and the activity was expressed as %. Activity lower than 10% is represented by “-“.

**Table 3:** Comparison of culture conditions before and after optimization.

Compared Factors	Before Optimization *	After Optimization
C/N source	NB	deCSP
Cultivation temperature (°C)	30	30
C/N Concentration (%)	0.8	1.6
Cultivation time (day)	4	4
Medium/flask volume ratio	100/250	130/250
Seed culture (%)	1	2
Inhibition (IC_50_ μg/mL)	81 ± 4.3	6.7 ± 0.31
aGI productivity (U/mL)	5000	12,400

* The optimal cultivation condition, the IC_50_ value and aGI productivity of FNB against *S. cerevisiae* α-glucosidase were obtained from a previous study [2].

**Table 4:** Specific inhibitory activity of fermented deCSP and acarbose against some enzymes.

Enzyme	Inhibition by deCSP		Inhibition by Acarbose	
	IC_50_ (µg/mL)	Max. Inh. Activity (%) *	IC_50_ (µg/mL)	Max. Inh. Activity (%) *
*S. cerevisiae* α-glucosidase	6.7 ± 0.31	99 ± 2.2	1095	74 ± 3.4 **
Rat intestinal α-glucosidase	15.9 ± 0.7	97 ± 2.7	78 ± 3.2	91 ± 3.1
*B. stearothermophilus* α-glucosidase	6.6 ± 0.22	95 ± 2.3	0.042 ± 0.003	99 ± 1.7
Rice α-glucosidase	6.7 ± 0.25	96 ± 1.9	3.04 ± 0.82	100 ± 2.1
Porcine pancreatic α-amylase	-	-	ND	ND
*B. subtilis* α-amylase	-	-	ND	ND

* Maximum inhibitory activity was tested at 125 µg/mL of FCSP or acarbose; ** Maximum inhibitory activity of acarbose against *S. cerevisiae* α-glucosidase was determined at 2500 µg/mL of acarbose. ND: Not determined; -: No activity.

## Supplementary Materials

The following are available online at www.mdpi.com/1660-3397/15/11/350/s1. Figure S1: The effect of some parameters on aGIs synthesis.
