# Supplementary material for: Reclamation of Marine Chitinous Materials for the Production of α-Glucosidase Inhibitors via Microbial Conversion
Source: Mar Drugs. 2017 Nov 7;15(11):350. doi: 10.3390/md15110350 (PMC5706040; doi:10.3390/md15110350)
Supplement: Supplementary file 1 [file marinedrugs-15-00350-s001.pdf]

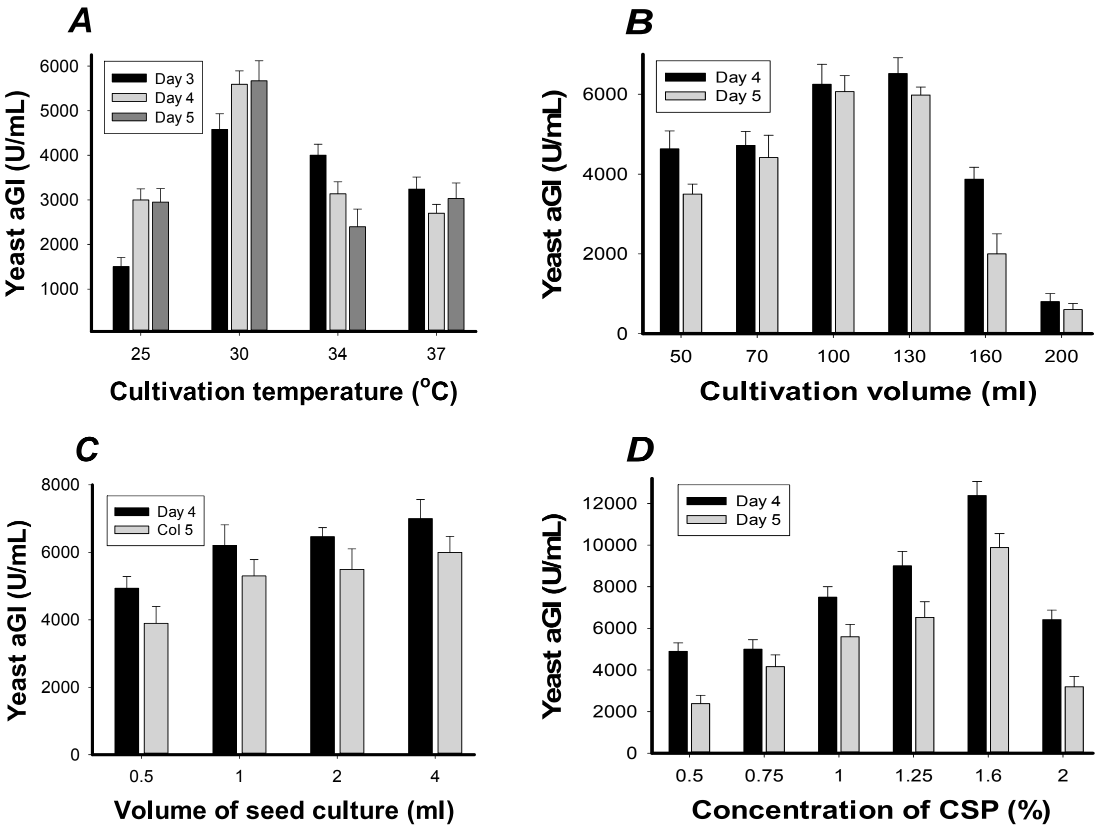

Figure S1. The effect of some parameters on aGIs synthesis. Cultivation temperature (**A**), medium culture volume (**B**), the inoculation amount of seed culture (**C**) and concentration of CSP (**D**). The experiment (**A**) was conducted at a temperature range of 25–37 °C, in 4–5 days (cultivation time), at 150 rpm (shaking speed), 100/250 mL (ratio volume of medium/flask), and bacterial seed solution of 1 mL (OD660nm = 0.25). The following experiments were designed based on the optimal conditions achieved from previous experiments. The *Saccharomyces cerevisiae* α-glucosidase inhibition of the supernatants was tested and expressed as U/mL.
